# *MiR-124-3p* impedes the metastasis of non-small cell lung cancer via extracellular exosome transport and intracellular PI3K/AKT signaling

**DOI:** 10.1186/s40364-022-00441-w

**Published:** 2023-01-04

**Authors:** Qing Zhu, Yixuan Zhang, Mo Li, Ying Zhang, Huan Zhang, Jiayi Chen, Zhaoyang Liu, Peng Yuan, Zhaogang Yang, Xiaobing Wang

**Affiliations:** 1 Department of Clinical Laboratory Diagnostics, Beijing Friendship Hospital, Capital Medical University, Beijing, 100050 China; 2 State Key Laboratory of Molecular Oncology, National Cancer Center/National Clinical Research Center for Cancer/Cancer Hospital, Chinese Academy of Medical Sciences and Peking Union Medical College, Beijing, 100021 China; 3 School of Life Sciences, Jilin University, No. 2699 Qianjin Street, Changchun, 130012 China; 4 Department of VIP Medical Services, National Cancer Center/National Clinical Research Center for Cancer/Cancer Hospital, Chinese Academy of Medical Sciences and Peking Union Medical College, Beijing, 100021 China

**Keywords:** *miR-124-3p*, Exosome, NSCLC, Metastasis

## Abstract

**Background:**

Metastasis is a significant factor that affects the survival of patients with non-small cell lung cancer (NSCLC). Nevertheless, the molecular regulatory mechanism underlying the metastasis is currently not fully understood. This study aims to identify the important role of *miR-124-3p* in metastasis of NSCLC, thereby providing a potential therapeutic intervention.

**Methods:**

Exosome secretion was determined by Nanoparticle Tracking Analysis (NTA) and the uptake was measured by fluorescence inverted microscope. The binding mechanism between *miR-124-3p* and its upstream or downstream target genes was validated experimentally by Luciferase reporter. Cells migration was evaluated by transwell assays. Transcriptome sequencing on A549 was carried out to verify the potential signaling pathway underlying *miR-124-3p* regulation. Western blotting analysis was used to assess the level of AKT, p-AKT, PI3K, and p-PI3K protein expression in NSCLC cell lines. The role of *miR-124-3p* to suppress the tumor metastasis was verified in NSCLC xenograft model.

**Results:**

Exosomes were more abundant in serum from patients with advanced lung cancer (*n* = 24 patients) than in these from patients with early-stage lung cancer (*n* = 30 patients), which suggested the potential correlation between amount of exosome secretion and the metastasis of NSCLC. Interestingly, the exosome release, uptake and the migration of NSCLC cells were notably inhibited by *miR-124-3p*. *LINC00511* suppressed the expression of *miR-124-3p* to facilitate exosome transport due to its role as the competitive endogenous RNA for *miR-124-3p*. The *miR-124-3p* could directly target the 3′-UTR of Rab27a in NSCLC cells to inhibit exosome secretion and thereby prevent cell migration and invasion. Aside from the inhibition of exosome transport, *miR-124-3p* inhibited the activation of PI3K/AKT signaling in the intracellular environment. Finally, by measuring subcutaneous tumor weight and volume and lung metastasis, we also demonstrated that *miR-124-3p* inhibited tumor growth in vivo.

**Conclusion:**

In NSCLC, *miR-124-3p* significantly suppressed metastasis through extracellular exosome transport and intracellular PI3K/AKT signaling. These findings provide new insights toward a better understanding of the NSCLC metastasis and suggest a potential treatment biomarker for NSCLC.

**Supplementary Information:**

The online version contains supplementary material available at 10.1186/s40364-022-00441-w.

## Background

Among all cancers, the incidence of lung cancer remains high, and it has the highest mortality rate of any malignant tumors in China in recent years [1, 2]. Of all lung cancers, 85% are non-small cell lung cancers (NSCLCs), and its rate of 5-year survival is extremely low [3]. Up to now, surgery, chemotherapy and radiotherapy are the main treatments of NSCLC. However, only less than half of the patients have the opportunity to receive radical surgical treatment, and the rate of 5-year survival is still remaining at 30–60% [4]. For the past few years, the treatment options of NSCLC have made some progresses, but local recurrence and distant metastasis are still the pivotal factors that affect the efficacy and survival of patients with NSCLC [5, 6]. Due to the relatively complex processes of metastasis regulation, there are no clinically significant predictive markers and targeted suppression methods for lung cancer invasion and metastasis [7]. Therefore, research for the key molecular mechanism underlying the invasion and metastasis has become an urgent requirement to improve the therapeutic effect of lung cancer, and has important practical significance for improving the prognosis of patients with NSCLC.

With intensive research into cancer, it was found that the microRNAs (miRNAs) have become special diagnostic and prognostic biomarkers of certain cancers, including prostate cancer, colorectal cancer, metastatic breast cancer, etc. [8–10]. Through binding to the 3’non-coding region (3’UTR), mature miRNAs can negatively regulate gene expression by degrading of the target mRNA or inhibiting its production [11, 12]. *MiR-124-3p* usually has an abnormal expression in several tumors. For example, in colorectal cancer cells, DNA synthesis and proliferation were inhibited by *miR-124-3p* through reduced expression of pentose phosphate pathway enzymes [13]. In bladder cancer, *miR-124-3p* targeted DNA methyltransferase 3B (DNMT3B) and downregulated its expression to suppress the capabilities of cell proliferation, invasion and migration [14]. It can also suppress tumor metastasis through repressing the expression of Foxq1 in nasopharyngeal carcinoma. As a potential biomarker, it can distinguish the patients in various clinical stages [15], suggesting *miR-124-3p* had a strong association with tumorigenesis, development, and outcome. Yet, the elaborate regulatory functions of *miR-124-3p* about metastasis in NSCLC is still not well characterized, and its regulatory mechanism is still unclear.

MiRNAs can also affect the tumor progression and prognosis by modulating the tumor microenvironment (TME). For example, *miR-146a* was upregulated in melanoma micro-environmental tissue and *miR-146a* could inhibit the level of *IFN-γ* by repressing the expression of STAT1, thus suppressing the abilities of melanoma proliferation and migration [16]. Overexpression of miR-148b-5p in gastric cancer also reprogrammed the metabolism profile, and the immune microenvironment was also modulated by *miR-148b-5p* through changing lymphocyte and myeloid cell populations [17]. As critical mediators of intercellular communication in TME, exosomes play vital function in the development of cancers. Compared with normal cells, tumor cells release more exosomes, which can promote tumor invasion and targeted metastasis in several tumor types [18, 19]. Several studies have demonstrated that high levels of exosome contents produced by tumor cells correlate significantly with poor prognosis in patients with tumor [20–22]. Besides, *miR-124a* can be packaged into exosomes in bone marrow-derived mesenchymal stromal cells (BM-MSCs) and showed anti-tumor effects [23], suggesting the association among *miRNAs*, exosomes and cancers.

Our research demonstrated that *miR-124-3p* could prevent cell metastasis via extracellular exosome transport and intracellular PI3K/AKT signaling pathway in NSCLC. These findings offer a better understanding of the metastasis in NSCLC and present a potential treatment for NSCLC.

## Methods

### Patient samples

Serum of 54 patients with NSCLC were collected from Cancer Hospital Chinese Academy of Medical Science, Beijing, China. The clinicopathological characteristics of the patients enrolled are summarized in Table S1. The specimens were all diagnosed by experienced pathologists and the serum was frozen at − 80 °C.

### Cell culture

We obtained A549, NCI-H1299, NCI-H460, NCI-H520 and NCI-H157 cells from Beijing’s National Infrastructure of Cell Line Resource and cultured them in RPMI 1640 medium containing 10% fetal bovine serum (FBS), 100 U/mL penicillin and 0.1 mg/mL streptomycin. The BEAS-2b cells were kindly donated by the Guangbiao Zhou’s research group and cultured in DMEM medium with 10% FBS and penicillin (100 U/mL)/streptomycin (0.1 mg/mL). All cells were cultured at 37 °C incubator with 5% CO_2_.

### Exosomes isolation

The total exosomes from serum were enriched with exosome isolation reagent (ThermoFisher Scientific; Waltham, MA, USA). 100 μl serum per patient was used for exosome extraction. Briefly, stored serum samples of patients were first thawed on ice, and then the samples were centrifuged for 30 minutes at the centrifugal force of 2000×g. The supernatant solution was then transferred into a new centrifugal tube and 0.2 volumes of exosome isolation reagent were added. Then, these samples were completely mixed with the reagent, incubated for 30 minutes on ice, and centrifuged for 10 minutes at the centrifugal force of 10,000×g. The pellet was resuspended in 100 μL PBS after careful removal of the supernatant. Nanoparticle Tracking Analysis (NTA) was used to measure the particle size and concentration of exosomes.

### Labeling exosomes

To examine the uptake of exosomes by NSCLC cells in vitro, exosomes were labeled using a PKH67 green fluorescent labeling kit (Sigma-Aldrich, MINI67) as previous described. In briefly, dilute the PKH67 linker stock solution 10 times with “Diluent C”, add the dye working solution to the exosomes, incubate for 10 min, then extract the exosomes again according to the exosome extraction method to remove the excess dye, take 200 μL of 1 × PBS to resuspend the precipitate, the precipitate is the stained exosomes. A549 and NCI-H1299 cells were incubated with the labeled exosomes at 37 °C for 24 hours. Images were acquired under confocal microscopy.

### SiRNA transfection

Each well of 6-well plates was seeded with 200,000 cells and cultured for 24 hours. 20 μM siRNA (GenePharma, Shanghai, China) were transfected into cells with RNAi Max (Life Technologies, ThermoFisher Distributer; Brendale QLD, Australia). Before use, the cells were washed three times with cold PBS.

### Cell viability analysis

3-(4,5)-dimethylthiahiazo(−z-y1)-3,5-di-phenytetrazoliumromide (MTT) assay was performed to evaluate the cell viability. The cells were seeded in 96-well plates (1000 cells/well), cultured for different days with different treatment. At the end of the incubation period, the cells were incubated with 1 mg/ml MTT solution. One hour later, the absorbance was measured at 450 nm and the data were assessed using a Bio-rad reader (Bio-Tek Instruments).

### Western blotting analysis

The extraction of total cellular protein was processed by lysis buffer (1 M Tris-HCL, pH 6.8, 10% SDS and 80% glycerin). Bicinchoninic acid (BCA) kit was used for the determination of protein concentration follow the manufacturer’s instruction. Briefly, 10% SDS-PAGE gel was applied for separating the 30 μg total protein, and then proteins were electrophoretically transferred to polyvinylidene fluoride (PVDF) membranes (Millipore; Burlington, MA, USA). In the subsequent step, the membranes were washed three times with Tris buffered saline+Tween (TBST) and blocked thoroughly with 5% skim milk. Incubation with primary antibodies was performed overnight on blocked PVDF membranes at 4 °C. Secondary antibodies were applied for 2 hours after primary antibody incubation. The detection of protein bands was verified with enhanced chemiluminescence kit (ECL; ThermoFisher Scientific, Waltham, MA, USA).

### RNA extraction and quantitative real-time PCR

In order to extract the total RNA, TRIzol was used according to manufacturer’s instruction (ThermoFisher Scientific, Waltham, MA, USA), and mRNA was reverse transcripted into cDNA using the PrimeScript RT Reagent Kit (TaKaRa; Tokyo, Japan). Then Quantitative real-time PCR (qPCR) was run with the SYBR® Premix Ex Taq™ (TaKaRa) on ABI QuantStudio5 (ABI; Indianapolis, IN, USA). Sequences of qPCR primers were presented in Table S2.

### Transwell assay

Cells were first digested into single cells and then resuspended in PBS at calculated density. 100 μL cell suspension without FBS was added to the upper chamber of the transwell, and 600 μL complete medium was added to the lower chamber. Then, cells were flushed twice with PBS after cultured for 48 hours and fix with 4% paraformaldehyde for 30 minutes. Next, 0.1% crystal violet was added to stain the cells for 10 minutes, and a cotton swab was used to lightly wipe away the upper layer of non-migrated cells and the migrated cells were further washed with cold PBS twice. Five visual fields were chosen to observe and count the cells under the microscope.

### Clone formation assay

Cells in the status of logarithmic growth were trypsinized and used to prepare the cell suspension. Cells were diluted to the calculated density and each well of 6-well plate was inoculated with 1000 cells. Cells were incubated for several days and monitored frequently. The culture process was terminated when visible clones were observed in the petri dish. Then, the culture medium in the wells was discarded, and adhered cells were washed several times. Next, 4% paraformaldehyde was used to fix cells for 30 minutes and 0.1% crystal violet was applied to stain cells for 10 minutes. Finally, the colony numbers were measured using Gel imaging analysis system (Syngene, GBOX-F3EE; Bangalore, India).

### Wound healing assay

Each well of 6-well plate was spread 200,000 cells and incubated overnight. Then, scribe perpendicular on each well with a 10 μL pipette tip. After the streaking was completed, cells were washed for several times and cultured in fresh medium without FBS. After 12 and 24 hours’ incubation, cells were observed under the microscope.

### Dual-luciferase reporter assay

The sequences of Rab27a 3′-WT-UTR (5′-UCAGAGCCAAAAGUGCCUUA-3′) and Rab27a 3′-MUT-UTR(5′-UCAGAGCCAAAACUCGCUUA-3′), hsa-*miR-124-3p*-WT (5′-UAAGGCACGCGGUGAAUGCC-3′) and hsa-*miR-124-3p*-MUT (5′-UUUCCGUGGCGGUGAAUGCC-3′) were synthesized by General Biosystems (Anhui, China) and inserted into the psiCHECK2 basic construct upstream of a luciferase reporter gene separately. Each well of 6-well plate was seeded with 200,000 cells, and cells were separately transfected with Rab27a 3′-UTR (wild or mutant type), *miR-124-3p* mimic or *miR-124-3p* inhibitor. The Promega’s Dual-Luciferase Reporter Assay System (E1910) was used to detect the sample luciferase activity.

### Transcriptome sequencing and analysis

The transcriptome sequencing of A549 cells transfected with mimic NC or *miR-124-3p* mimic were performed by Majorbio (Shanghai, China). The RNA library preparation and deep-sequencing were performed based on instructions. The mRNA profiles were characterized by R program and related packages. Enrichment pathway analyzes were conducted with Kyoto Encyclopedia of Genes and Genomes (KEGG) (http://www.genome.jp/kegg/) and Gene Ontology (GO) database (http://www.geneontology.org).

### Xenograft model

A549 cells which were previously transfected with mimic control or *miR-124-3p* mimic were injected into the BALB/C nude mice subcutaneously (Beijing HFK Bioscience CO. Ltd.; Beijing, China). By the time tumor volume reached 100 mm^3^, the tumor information was measured manually every 3 days and the total volume was calculated as (a × b^2^)/2 (a = longest length of diameter, b = shortest length in diameter). Mice were sacrificed when the tumor volume reached 500 mm^3^. In another experiment, BALB/C nude mice were subcutaneously inoculated with 200,000 A549-Luc tumor cells via the tail vein. After intraperitoneal injection of D-luciferin (150 mg/kg), mice were monitored with bioluminescence imaging (BLI) of lung metastatic tumors in mice using an in vivo IVIS Lumina XRMS Series III imaging system (PerkinElmer; Richmond, CA, USA).

### Statistical analysis

The data were shown as the mean ± SD. The two-tailed unpaired student’s t test was used to assess statistical significance where *P* < 0.05 was considered to be significant. The data were analyzed using GraphPad Prism6 software (San Diego, CA, USA).

## Results

### Exosome is enriched in the serum of patients with metastatic NSCLC

**Fig. 1:**
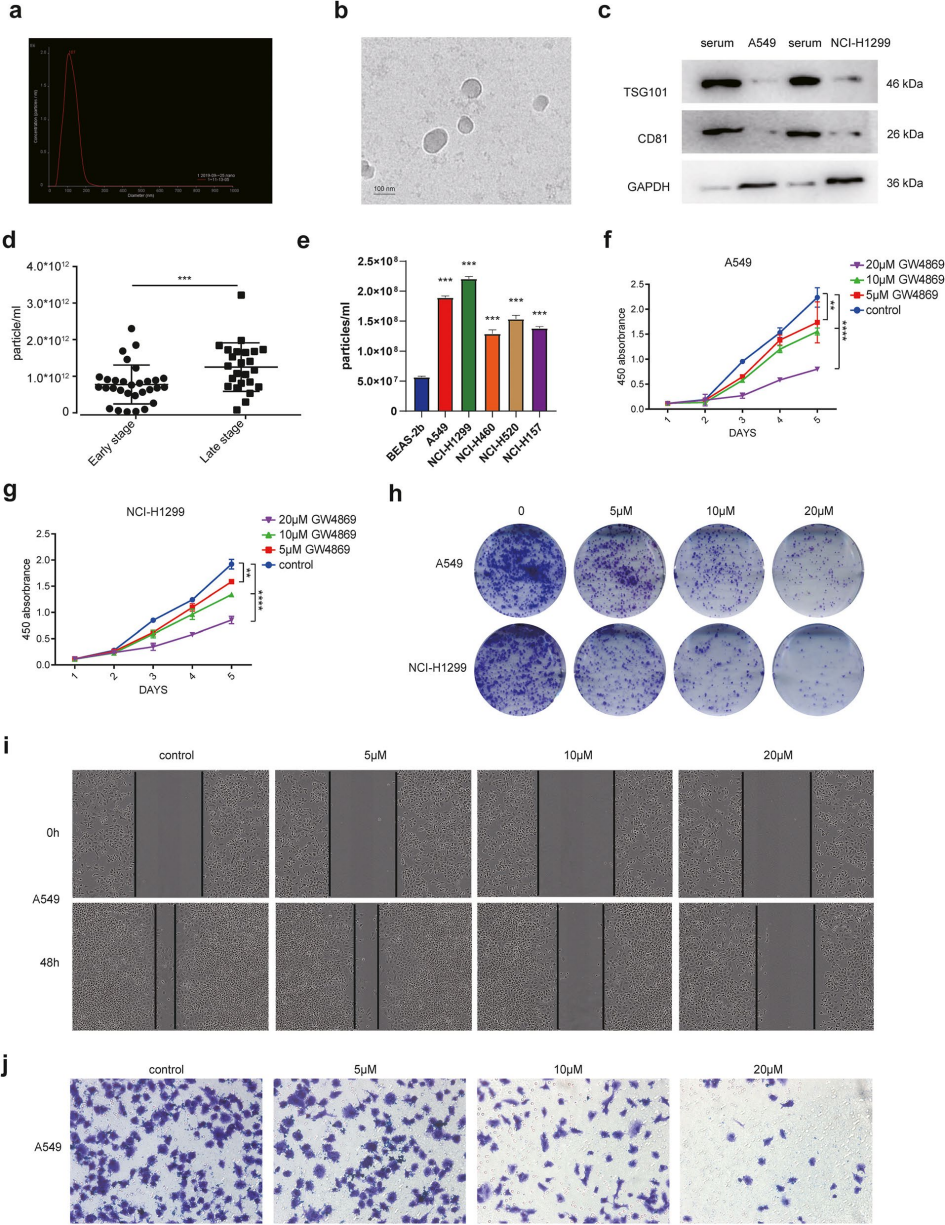
The release of exosomes affects tumor invasion and migration. **a** Size measurement of exosomes from NSCLC patient serum samples by NTA. **b** Image of exosomes captured with Cryo-TEM. Scale bar, 100 nm. **c** Images of Western blot analysis to detect the expression of exosomal markers, TSG101 and CD81, in vesicles isolated from NSCLC patient serum samples and NSCLC cells. Full-length blots are presented in Additional file 3. **d** Exosome concentration in serum of early and late-stage NSCLC patients. **e** Exosome concentration of NSCLC cell lines and normal lung epithelium cell line in culture. **f** Growth curve of A549 cells treated with different concentrations of an inhibitor of exosome release, GW4869. **g** Growth curve of NCI-H1299 cells treated with different concentrations of GW4869. **h** Images of colony forming assays for A549 and NCI-H1299 cells treated with GW4869. **i** Images of wound healing assays for A549 treated with GW4869. **j** Transwell assays performed with A549 treated with GW4869. Data are shown as the mean ± SD. *P < 0.01, **P < 0.001, ***P < 0.0001, and ****P < 0.00001

To explore the function of exosomes in NSCLC, we collected serum from 24 cases of advanced lung cancer (Stage III-IV) and 30 cases of early lung cancer (Stage I-II) (Table 1). The serum exosomes were isolated and NTA results revealed that the particles size obtained was typical of exosomes which were 50-120 nm in diameter (Fig. 1a). In addition, Cryo- transmission electron microscope (TEM) revealed that the predominant vesicles were of typical exosomal size and shape (Fig. 1b). Western blot analysis indicated the isolated vesicles from serum contained higher level of exosomal markers including CD81, and TSG101, compared with NSCLC cells (Fig. 1c). The exosome numbers in the serum of patients with advanced lung cancer (most of whom have the lymph node metastasis) were remarkably higher than that in patients with early-stage lung cancer (Fig. 1d). Meanwhile, the exosome number was correlated positively with the clinical staging of patients (Table 1). Also, we found that the secretion of exosomes in BEAS-2b cells (which is a normal lung epithelium) was significantly lower than that in NSCLC tumor cell lines (Fig. 1e). Interestingly, when we blocked exosome secretion in NSCLC cells by GW4869, a drug that hinders exosome biogenesis, the cell proliferation was dramatically reduced as determined by MTT and clone formation (Figs. 1f-h). Meanwhile, the proliferative capacity of NSCLC cells treated with both GW4869 and exosome was restored compared to GW4869 alone, indicating that the exosome depletion by GW4869 leads to cell inhibition (Fig. S1a). Moreover, GW4869 also inhibited the tumor invasion and migration at a dose-dependent manner in both A549 and NCI-H1299 (Figs. 1i, j, Fig. S1b, c). These results demonstrated that exosome numbers are increased in the serum of advanced patients with NSCLC, and exosomes could promote tumor cell proliferation and metastasis.

**Table 1:** Exosome concentration and clinicopathological characteristics of NSCLC

Variable	Number (%)	Exosome concentration (particle/ml)	Total	***P***-value
**Age No.**				
> 60	18(33.3)	7.63794E+ 11	54	**0.4328**
≤ 60	36(66.7)	8.92556E+ 11		
**Gender No.**				
Male	18(33.3)	9.95706E+ 11	54	**0.1795**
Female	36(66.7)	7.766E+ 11		
**Histology**				
Squamous	24(44.4)	8.10958E+ 11	54	**0.6555**
Adenocarcinoma	30(55.6)	8.80577E+ 11		
**Clinical stage**				
I/II	30(55.6)	4.90977E+ 11	54	**< 0.0001**
III/IV	24(44.4)	1.29796E+ 12		****

### *miR-124-3p* impedes the exosome secretion, uptake and migratory abilities of NSCLC cells

**Fig. 2:**
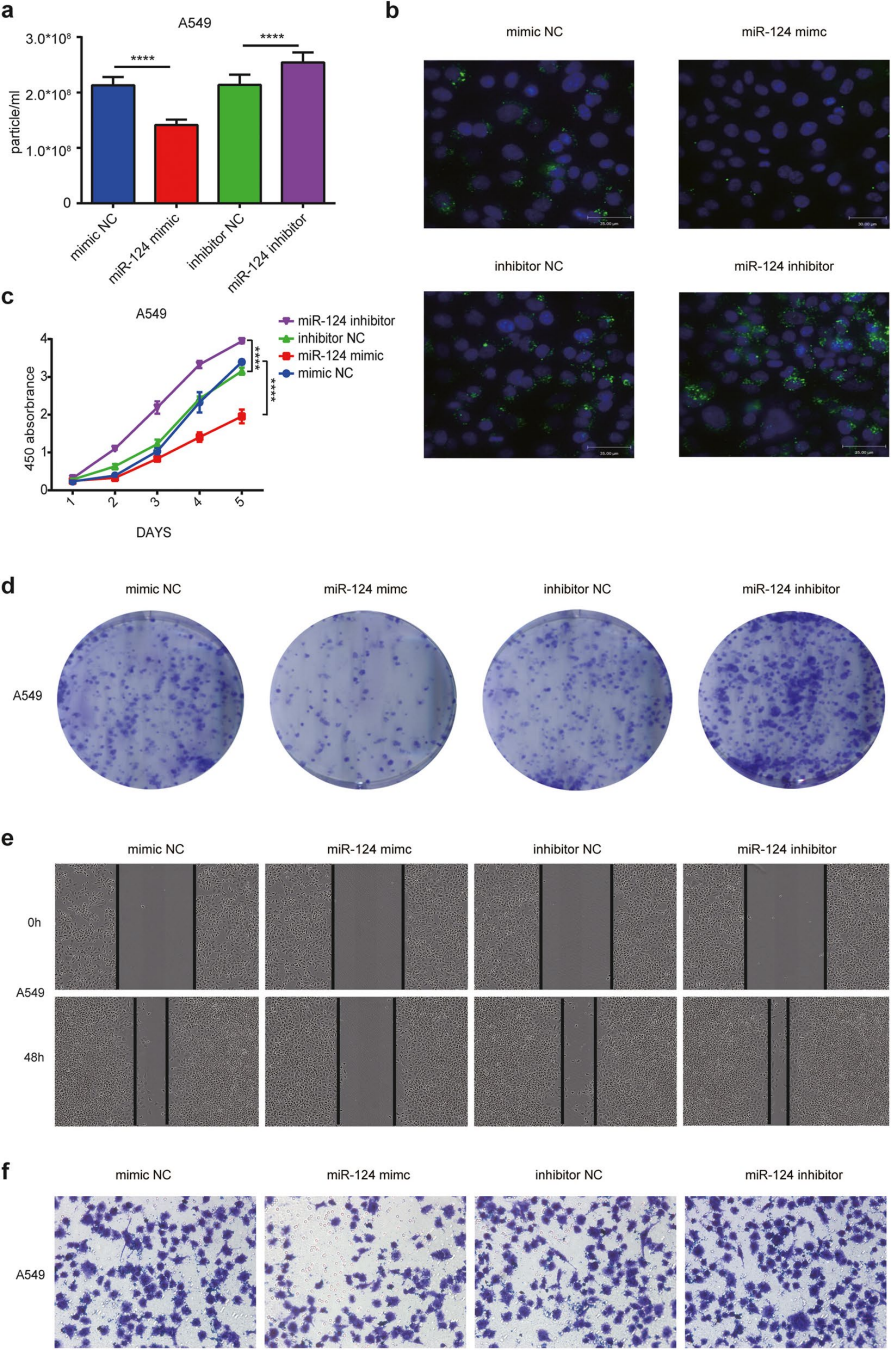
*miR-124-3p* inhibits exosome secretion, uptake and migratory abilities of NSCLC cells. **a** Concentration of exosomes in medium from A549 cells transfected with *miR-124-3p* mimic or inhibitor. **b** Images of the uptake of exosomes fluorescently labeled with PKH67 (green) by A549 after incubation for 24 hours. Images were acquired under confocal microscopy. **c** Growth curves generated with OD450 data with the CCK8 assay for A549 cells transfected with miR-124-3p mimic or inhibitor. **d** Images of colony forming assays for A549 cells transfected with *miR-124-3p* mimic or inhibitor. Cells were fixed and stained with crystal violet. **e** Images of wound healing of A549 cells transfected with miR*-124-3p* mimic or inhibitor. **f** Transwell assays performed with A549 cells transfected with *miR-124-3p* mimic or inhibitor. Cells were fixed and stained with crystal violet. Data are shown as the mean ± SD. *P < 0.01, **P < 0.001, ***P < 0.0001, and ****P < 0.00001

The secretion of exosomes is closely associated with the expression of various microRNAs [24, 25], and decreased expression of *miR-124-3p* is the indication contributing to the metastasis in many types of tumors [26–28], suggesting *miR-124-3p* might be related to the secretion of exosomes to influence the tumor migration and invasion. To corroborate whether *miR-124-3p* actually affects the release of exosomes, *miR-124-3p* mimic or inhibitors were transfected into NSCLC cells and the exosome release from cells after transfection was characterized. Results revealed that *miR-124-3p* inhibitors significantly promoted the exosome release while its mimic dramatically inhibited this process in NSCLC cells (Fig. 2a, Fig. S2a). Next, we labeled exosome with PKH67 green fluorescence to check the *miR-124-3p* effect on the cellular uptake of exosome in NSCLC cells. The co-application of *miR-124-3p* mimic with the PKH67-labeled exosomes to the A549 greatly inhibited the cellular uptake of the exosomes. Conversely, *miR-124-3p* inhibitor could enhance the exosome uptake into cells in A549 (Fig. 2b). Similar trends were observed in NCI-H1299 cell line (Fig. S2b). Furthermore, to enhance our understanding of the biological functions of *miR-124-3p* in NSCLC, cells transfected with *miR-124-3p* mimic or inhibitors were assessed in proliferation, migration and invasion assays. The growth curves generated with OD450 values from the MTT test and the colony formation indicated that *miR-124-3p* inhibited tumor cell proliferation in culture. Conversely, the *miR-124-3p* inhibitor could increase the ability of tumor cell growth in culture (Figs. 2c, d; Fig. S2c, d). Cell wound scratch assay revealed that *miR-124-3p* mimic notably inhibited NSCLC cell invasion, while transfection with *miR-124-3p* inhibitors showed the reverse trend (Fig. 2e, Fig. S2e). Meanwhile, the down-regulation of *miR-124-3p* in NSCLC cells promoted migratory abilities of the NSCLC cells, as detected by transwell assay (Fig. 2f, Fig. S2f).

### *miR-124-3p* is down-regulated by its ceRNA-*LINC00511*

**Fig. 3:**
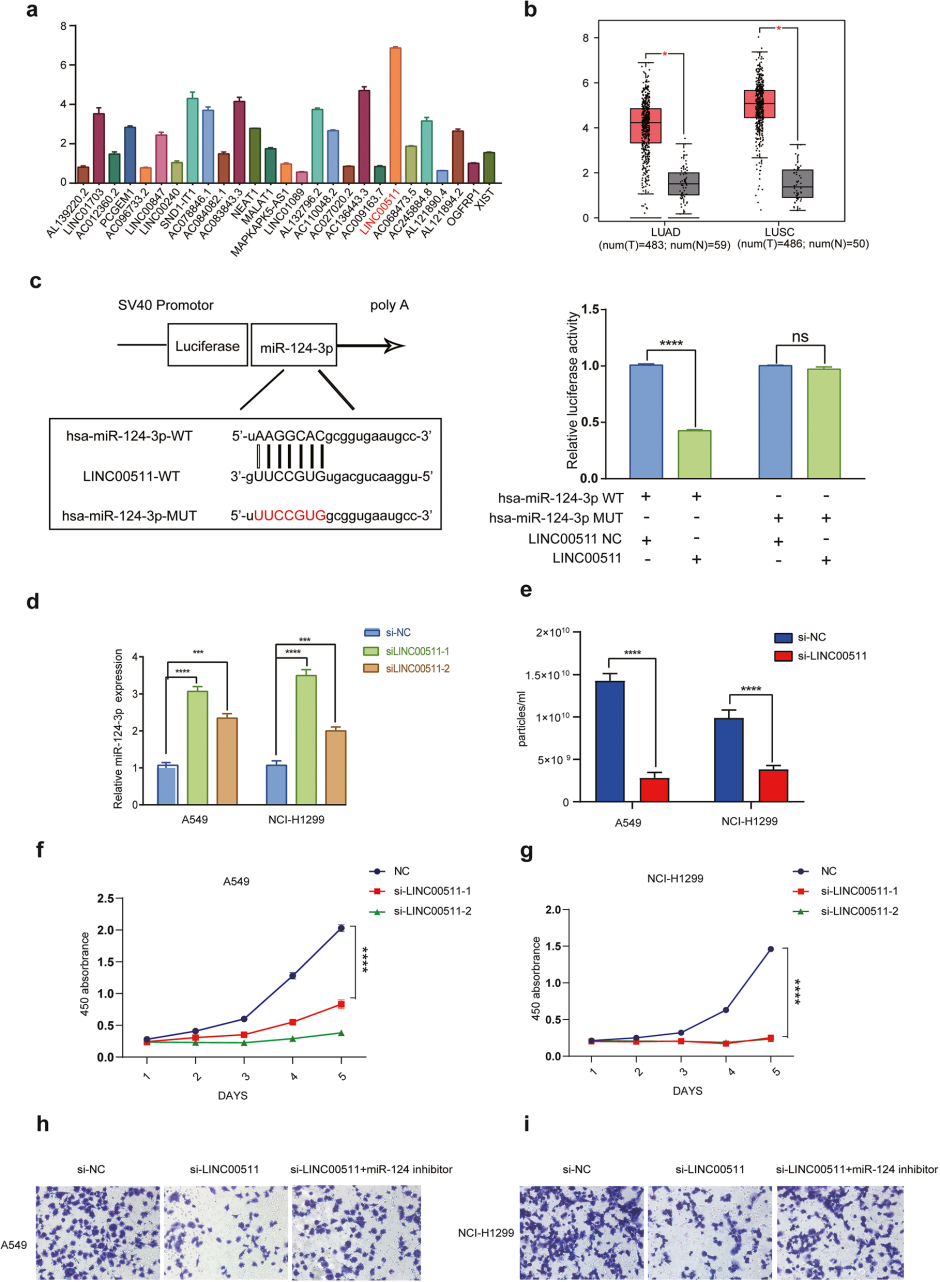
*miR-124-3p* is down-regulated by *LINC00511*. **a** The relative RNA expression of different LncRNA in A549 cells. **b** The expression of *LINC00511* in NSCLC by analyzing the TCGA dataset using the GEPIA database. LUAD means Lung Adenocarcinoma, LUSC means Lung Squamous Carcinoma. Red box means tumor, gray box means normal tissue. **c** Luciferase activity from NSCLC cells transfected with *LINC00511* and luciferase reporter constructs containing WT-*miR-124-3p* or MUT-*miR-124-3p* binding sequences for *LINC00511*. **d** The relative expression of *miR-124-3p* in A549 and NCI-H1299 cells with *LINC00511* knockdown. **e** Exosome concentration in medium from A549 and NCI-H1299 cells transfected with siRNA against *LINC00511*. **f** Growth curves generated with OD450 data with the CCK8 assay for A549 cells with *LINC00511* knockdown. **g** Growth curves generated with OD450 data with the CCK8 assay for NCI-H1299 cells with *LINC00511* knockdown. **h** Transwell assays performed with A549 cells transfected with si-*LINC00511* or si-*LINC00511* + *miR-124-3p* inhibitor. **i** Transwell assays performed with NCI-H1299 cells transfected with si-*LINC00511* or si-*LINC00511* + *miR-124-3p* inhibitor. Data are shown as the mean ± SD. *P < 0.01, **P < 0.001, ***P < 0.0001, and ****P < 0.00001

Recent research reported that the interaction between lncRNA and miRNA can modulate the expression of downstream genes and affect different biological functions [29–32]. To identify lncRNA that regulate *miR-124-3p* signaling, we used starBase v2.0 software to predict the lncRNA-miRNA interaction. From the prediction, a total of 33 potential lncRNAs were chosen, and qPCR was used to check their expression. Among them, *LINC00511* was found to have the highest expression in the NSCLC cells tested (Fig. 3a) and Gene Expression Profiling Interactive Analysis (GEPIA) further manifested that *LINC00511* expression was remarkably upregulated in lung cancer tissues compared with normal tissues (Fig. 3b). Dual-luciferase reporter research demonstrated that *LINC00511* could suppress *miR-124-3p* mimic driven luciferase activity, while the suppression by *LINC00511* was abolished with the binding site mutated (Fig. 3c). Furthermore, knockdown of *LINC00511* significantly increased *miR-124-3p* expression, suggesting the negative regulation of *LINC00511* as a competing endogenous RNA (ceRNA) of *miR-124-3p* (Fig. 3d). Meanwhile, we selected 10 NSCLC patients with paired pathological tissues for RNA extraction and RT-PCR. The results showed that the expression of *LINC00511* was significantly higher in tumor tissues than that in normal tissues, while the expression of *miR-124-3p* was the opposite (Fig. S3a). The correlation index of two gene expression levels is − 0.79, *P* = 0.0069 (Fig. S3b), indicating that the expression of *LINC00511* was negatively correlated with *miR-124-3p* in NSCLC. With the *LINC00511* knockdown, exosomes secretion was inhibited compared to the control (Fig. 3e). Finally, we examined the impacts of *LINC00511* on the progression of NSCLC. Obviously, the capabilities of cell proliferation and migration were significantly reduced after *LINC00511* was knocked down. However, the co-culture with the *miR-124-3p* inhibitor and *LINC00511* partially rescued the phenotype (Fig. 3f-i). These results indicated that *LINC00511* served as the ceRNA to repress the expression of *miR-124-3p* and enhance the progression of NSCLC through the exosomes.

### *miR-124-3p* regulates the exosome production by targeting Rab27a

**Fig. 4:**
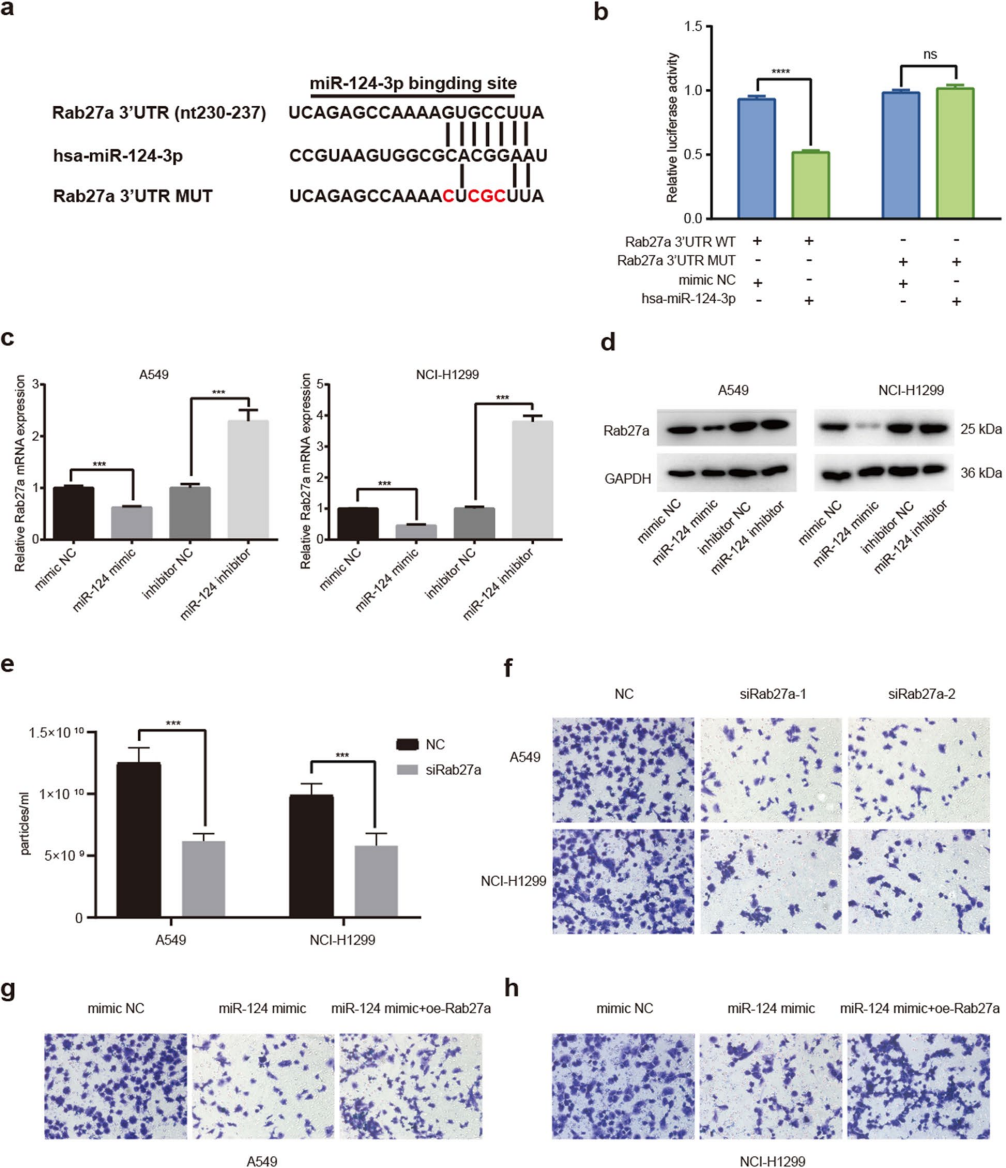
*miR-124-3p* inhibits tumor metastasis by targeting Rab27a. **a** Sequence alignment of *miR-12-3p* with WT-Rab27a-3′-UTR and MUT- Rab27a-3′-UTR. The mutant sites were colored in red. **b** Luciferase activity from cells transfected with *miR-124-3p* and luciferase reporter constructs containing the WT-3-UTR or MUT-3-UTR of Rab27a. **c** qPCR to detect expression of Rab27a in A549 and NCI-H1299 cells transfected with *miR-124-3p* mimic, inhibitor, or mimic NC (control). **d** Western blot to detect protein expression of Rab27a in A549 and NCI-H1299 cells transfected with *miR-124-3p* mimic, inhibitor or mimic NC (control). Full-length blots are presented in Additional file 3. **e** Exosome number in medium from A549 and NCI-H1299 cells transfected with siRNA against Rab27a, si-Rab27a. **f** Transwell assays performed on A549 and NCI-H1299 cells transfected with si-Rab27a. **g** Transwell assays performed in A549 cells transfected with *miR-124-3p* mimic or *miR-124-3p* mimic + oe-Rab27a. **h** Transwell assays performed in NCI-H1299 cells transfected with *miR-124-3p* mimic or *miR-124-3p* mimic + oe-Rab27a. Data are shown as the mean ± SD. *P < 0.01, **P < 0.001, ***P < 0.0001, and ****P < 0.00001

To find putative target genes of *miR-124-3p* that regulate the exosome production, prediction algorithms TargetScan was performed to predict the downstream genes of *miR-124-3p.* Rab27a is a member of RAS oncogene superfamily, Rab27a silencing inhibits exosome secretion without modifying exosome protein composition [33]. It was determined as a potential target gene of *miR-124-3p* (Fig. 4a). The dual-luciferase reporter test demonstrated *miR-124-3p* mimic apparently repressed the luciferase activity of the Rab27a wild-type 3′-UTR reporter compared with the negative control, while the luciferase activity of the Rab27a mut-type 3′-UTR reporter was unaffected by *miR-124-3p* mimic or inhibitors, indicating that *miR-124-3p* inhibits Rab27a expression via directly targeting its 3′-UTR (Fig. 4b). Through qPCR and Western blotting analysis, we substantiated that *miR-124-3p* mimic decreased both the Rab27a mRNA and protein level in NSCLC cell lines (Figs. 4c, d). Since Rab27a can be targeted by *miR-124-3p*, we determined the biological functions of Rab27a in the exosome production. With the Rab27a knockdown by its siRNA, exosomes secretion was significantly inhibited as compared with control group (Fig. 4e). Moreover, the decreased cell proliferation and metastasis was observed with the Rab27a knockdown in NSCLC cells as characterized by clone formation and transwell assays (Fig. 4f, Fig. S4a), and this phenotype is very similar to *miR-124-3p* treatment group. Interestingly, we found that the abilities of cell clone formation and migration were restored with the re-expression of Rab27a in cells transfected with *miR-124-3p* mimic (Figs. 4g, h, Fig. S4b). These results indicated that *miR-124-3p* had a function of inhibiting the metastasis of NSCLC through suppressing the translation of Rab27a.

### *miR-124-3p* inhibits lung cancer progression through PI3K/AKT signaling pathway

**Fig. 5:**
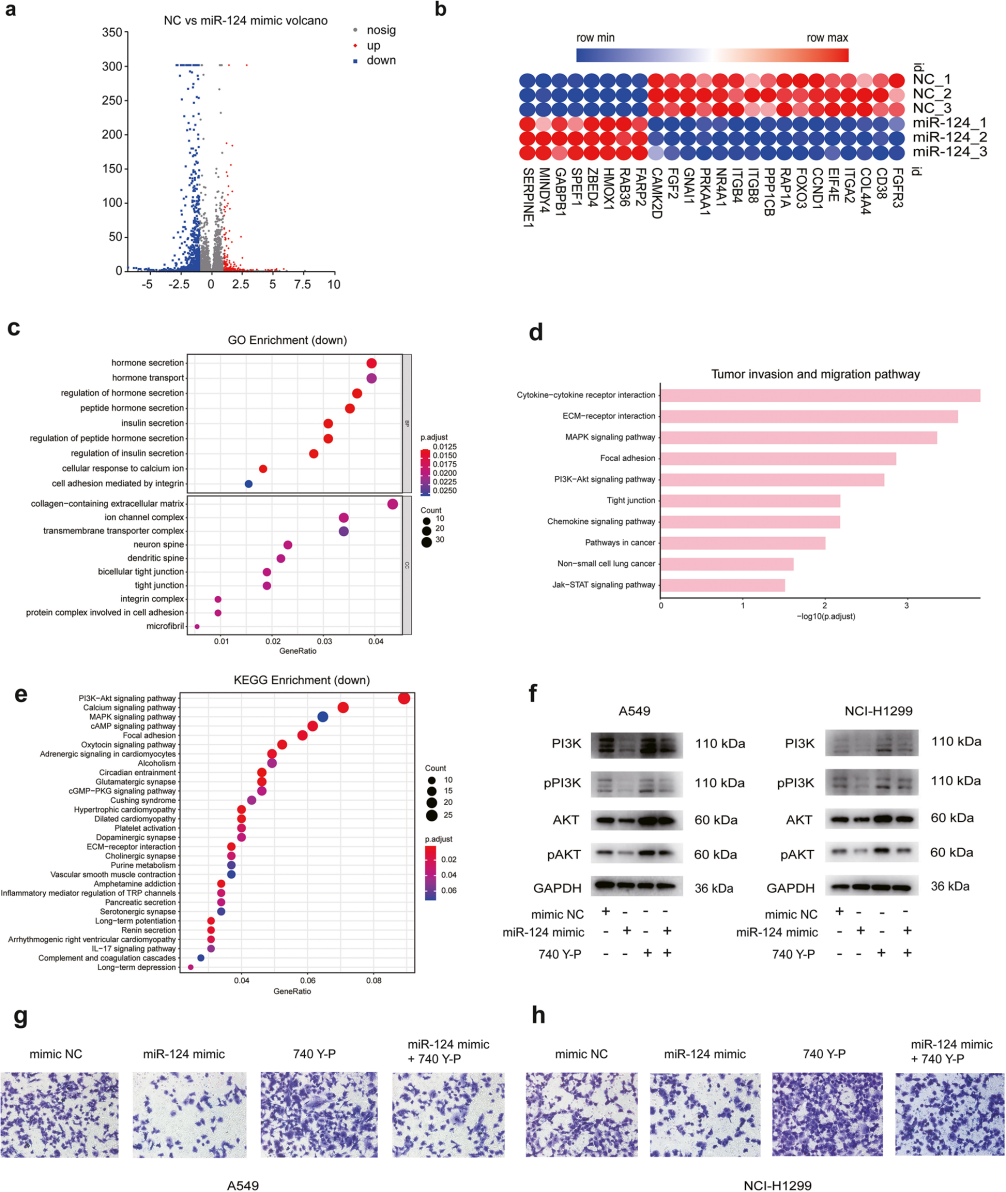
*miR-124-3p* inhibits NSCLS progression partially through PI3K/AKT signaling pathway. **a** Volcano plot presenting differentially expressed genes with *miR-124-3p* mimic transfection. Blue dots represent the downregulated genes (fold change < 0.5, FDR ≤ 0.05) and red dots represent the upregulated genes (fold change > 2, FDR ≤ 0.05). **b** The heatmap of differentially abundant genes obtained from the mimic NC and *miR-124-3p* mimic groups. **c** Significantly downregulated GO pathways with *miR-124-3p* mimic transfection. **d** The tumor invasion and migration pathway KEGG pathways enrichment in *miR-124-3p* mimic groups. **e** Significantly downregulated KEGG pathways with *miR-124-3p* mimic transfection. **f** Western blot to detect protein expression of AKT, p-AKT, PI3K and p-PI3K in A549 and NCI-H1299 cells transfected with mimic NC, *miR-124-3p* mimic,740 Y-P (20 μM) or *miR-124-3p* mimic+ 740 Y-P (20 μM). Full-length blots are presented in Additional file 3. **g** Transwell assays performed in A549 cells transfected with *miR-124-3p* mimic, 740 Y-P (20 μM) or *miR-124-3p* mimic + 740 Y-P (20 μM). **h** Transwell assays performed in NCI-H1299 cells transfected with *miR-124-3p* mimic, 740 Y-P (20 μM) or *miR-124-3p* mimic + 740 Y-P (20 μM)

To further investigate the regulatory mechanism underlying *miR-124-3p*-regulated lung cancer progression, we performed transcriptome sequencing on A549 cells transfected with mimic NC or *miR-124-3p* mimic. 213 genes were differentially up-regulated (fold change > 2, FDR ≤ 0.05) and 908 genes were differentially down-regulated (fold change < 0.5, FDR ≤ 0.05) in NSCLC cells transfected with *miR-124-3p* mimic compared with control (Fig. 5a, Table S3). Among these genes, several genes related to tumor invasion and migration were down-regulated (Fig. 5b). The genes decreased expression levels were enriched in GO terms related to hormone secretion, collagen-containing extracellular matrix processes (Fig. 5c). Many pathways related to tumor invasion and migration are significantly enriched and *miR-124-3p* has been proved to play a critical role in tumor invasion and migration (Fig. 5d). Moreover, KEGG enrichment analysis showed that the phosphatidylinositol-3-kinase/AKT (PI3K/AKT) signaling pathway was significantly down-regulated (Fig. 5e). Due to the importance of PI3K/AKT signaling pathway in cancer development, we performed Western blotting analysis on NLCSC cells. The cells were divided into four groups including control, treated with *miR-124-3p* mimic alone, treated with PI3K activator 740 Y-P (20 μM) alone and treated with combination of *miR-124-3p* mimic and PI3K activator 740 Y-P. The results showed that the expression of PI3K, p-PI3K, AKT, p-AKT was decreased treated with *miR-124-3p* mimic alone compared to the NC group, the expression of these proteins was increased treated with 740 Y-P alone. Interestingly, we found that the expression of these proteins was restored with the re-expression of *miR-124-3p* in cells treated with 740 Y-P (Fig. 5f). Furthermore, the ability of cell invasion was diminished in the *miR-124-3p* mimic alone group and enhanced in the PI3K activator alone group compared to the control group. Compared with the PI3K activator alone group, the ability of cell invasion was diminished in the combination of *miR-124-3p* mimic and PI3K activator 740 Y-P, which suggests that *miR-124-3p* can inhibited the PI3K signaling pathway (Figs. 5g, h). These results confirmed the *miR-124-3p* inhibited lung cancer progression in part via intracellular PI3K/AKT signaling pathway.

### *miR-124-3p* inhibits lung metastasis of NSCLC in vivo

**Fig. 6:**
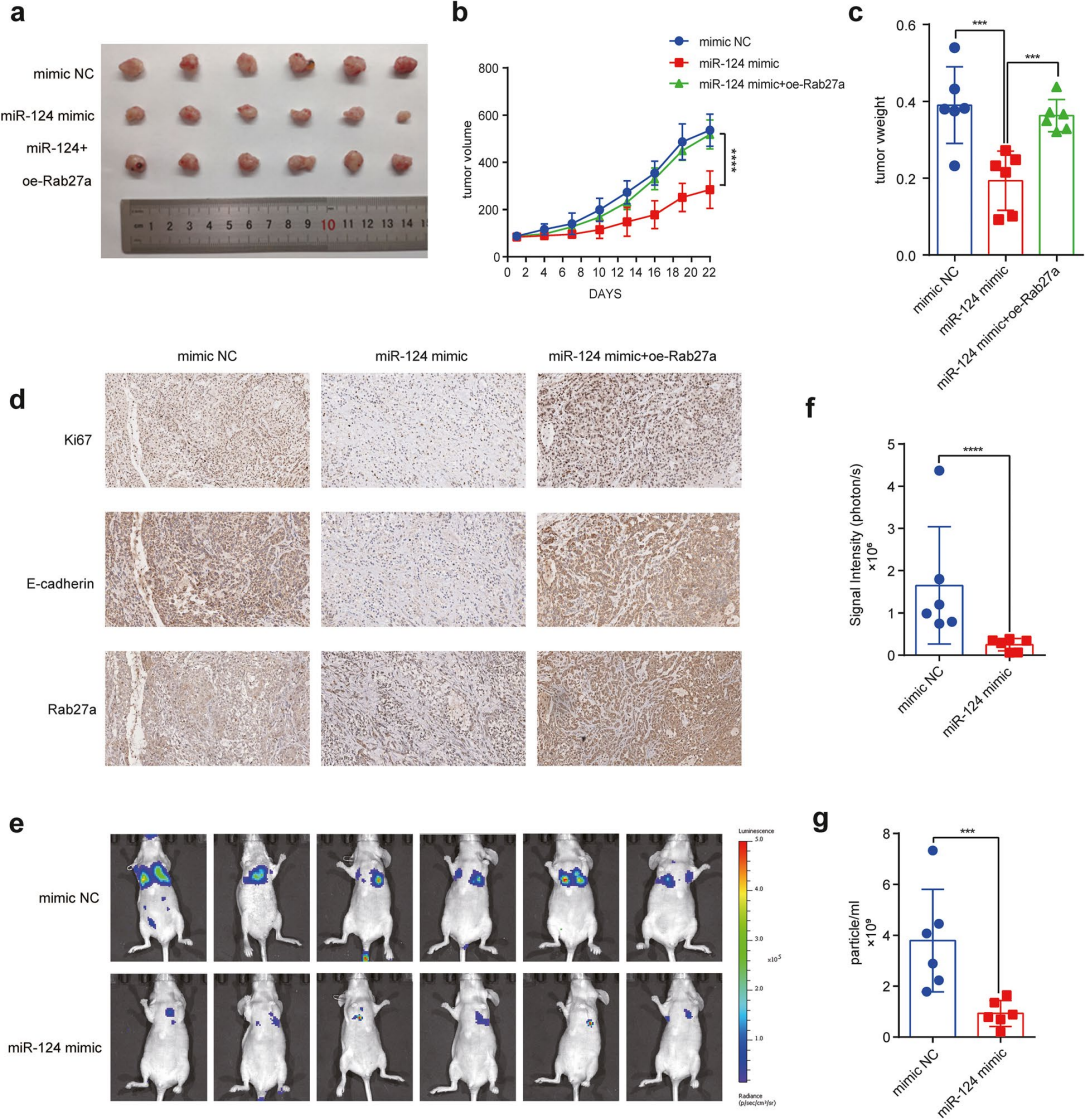
*miR-124-3p* inhibits tumor growth and metastasis in vivo*.* **a** Representative images of xenograft tumors derived from A549 cells expressing mimic NC, *miR-124-3p* mimic or *miR-124-3p* mimic + oe-Rab27a implanted in nude mice. **b** Quantification of xenograft tumor volume at different days after tumor volume reached 100 mm^3^ **c** Weight of tumors harvested from animals after 22 days of monitoring. **d** Images of immunohistochemical staining for Ki67, E-cadherin and Rab27a in xenograft tumors. **e** Bioluminescence images of lung metastases generated with A549 cells expressing mimic NC, *miR-124-3p* mimic or *miR-124-3p* mimic + oe Rab27a. **f** Quantification of bioluminescence from the lung metastases. **g** Concentration of exosomes in plasma of nude mice implanted with modified A549 cells. Data are shown as the mean ± SD. *P < 0.01, **P < 0.001, ***P < 0.0001, and ****P < 0.00001

Since *miR-124-3p* modulated the ability of proliferation of NSCLC in vitro, we further evaluated its function on tumor growth in vivo. Firstly, NSCLC cells were transfected with different agents including mimic NC, *miR-124-3p* mimic and *miR-124-3p* mimc+overexpressed-Rab27a (oe-Rab27a), and then subcutaneously injected into nude mice. The tumor growth was assessed using the tumor volume and mice weight accurately measured every 3 days. Tumor growth of *miR-124-3p* mimic transfecting group was significantly reduced compared to the mimic NC transfecting group, and the re-expression of Rab27a restored tumor growth (Figs. 6a-c). Immunohistochemistry (IHC) was applied to evaluate the protein expression of Ki67, E-cadherin and Rab27a, confirming the lower expression of these two proteins in *miR-124-3p* mimic group compared to the control and *miR-124-3p* mimc+oe-Rab27a group (Fig. 6d). Since the low-level of *miR-124-3p* enhance the release of exosomes to promote the metastasis of NSCLC cells, so we construct the lung metastasis model to evaluate the impact of expression of *miR-124-3p* on lung metastasis. We generated A549 cells stably overexpressing *miR-124-3p* by infecting the *miR-124-3p* mimic lentivirus and these cells with *miR-124-3p* overexpression were injected into lateral tail veins. Our results revealed that the growth of lung metastatic lesions was markedly inhibited compared to the control group (Figs. 6e, f). Further, we collected the serum of mice and found that the exosomes number in the serum were indeed reduced with the *miR-124-3p* mimic (Fig. 6g). These results further indicated that *miR-124-3p* can regulate the release of exosomes to promote the development of NSCLC.

## Discussion

Exosomes are small vesicles with a diameter of 30–100 nm, and they contain some functional biomolecules including DNA, RNA, lipids and proteins, which can be transferred to recipients. Study showed that tumor-derived exosomes prepared a favorable microenvironment at the site of future metastasis and contribute to a non-random metastatic pattern [34]. Researches on exosome have focused on the regulatory function of their contents on cancer occurrence and metastasis, with the exosomal miRNA have become the hotspot of research. For example, tumor-derived exosome miR-934 was shown to induce macrophage M2 polarization and promote liver metastasis in colorectal cancer [35]. In our study, it is the first time to demonstrate that high-level exosome is corrected with the high-level clinical staging of patients, and tumor-derived exosomes could promote tumor cell proliferation and metastasis.

As an exosomal content, *miR-124-3p* has been extensively investigated in nervous system, where the neuron-specific *miR-124-3p* has the potential to be immobilized in *miR-124-3p* into astrocytes and upregulates the major glutamate transporter GLT1 by inhibiting microRNAs that suppress GLT1 [36]. Moreover, the abnormal expression of *miR-124-3p* has been linked to the occurrence and development of various malignant tumors. In ovarian cancer, *miR-124-3p* is suppressed by circ_0026123 leading to the cell progression, thereby functions as a tumor suppressor [37]. *MiR-124-3p* is also down-regulated in colorectal cancer, and it can target the 3’UTR of PD-L1 and reduce its translation resulting in remarkable antitumor effects. It also reduced the expression of MMP-9 and subsequently inhibited cell migration and invasion in CRC [38]. In our research, we first reported that *miR-124-3p* inhibits the exosome secretion and migratory abilities of NSCLC cells through targeting Rab27a and another function of *miR-124-3p*-mediated lung cancer progression indicated that PI3K/AKT signaling pathway was regulated by *miR-124-3p*. This is the first time to illuminate the functions of *miR-124-3p* in cancer metastasis from the extracellular and intracellular perspective.

In some studies, Rab27a is demonstrated to regulate exosome production and metastasis. Disrupting Rab27a expression reduces exosome release, tumor growth, and metastasis in melanoma, pancreatic cancer and colorectal cancer [39–41]. In our study, we demonstrated that *miR-124-3p* inhibits Rab27a expression via directly targeting its 3′-UTR, which inhibiting the exosome secretion. Furthermore, increasing evidence suggests lncRNA participates in the regulation of target miRNA as a ceRNA [42]. The extensive network of lncRNA-miRNA-mRNA interactions mediates a variety of biological functions in cancer, including DNA damage, epigenetic regulation, cell cycle regulation, involvement in signal transduction pathways and hormone-induced cancer [43–45]. In our research, we demonstrated *miR-124-3p* was down-regulated by *LINC00511*, and silencing *LINC00511* could inhibit the secretion of exosome, cell proliferation and migration in NSCLC. These results identified the microRNA, mRNA and lncRNA interactions and thus a potential feature of *miR-124-3p* in the progression and metastasis of NSCLC.

Although we discovered *LINC00511/miR-124-3p/ Rab27a* axis which regulates the secretion of exosome in NSCLC metastasis and the detailed regulatory function of *miR-124-3p* in PI3K/AKT signaling pathway, our research still has some shortages. For example, the number of clinical samples was limited while the result of exosome enrichment in the metastatic NSCLC was based on 54 patients. Therefore, a large-scale clinical validation has to be conducted to further verify our findings. Then, the detailed mechanism between *LINC00511* and *miR-124-3p* is still not clear, and further investigation is needed in our future research. Besides, although we have found the role of *miR-124-3p* from extracellular and intracellular aspects, we have not demonstrated whether there is feedback regulation between exosomes release in the microenvironment and the PI3K/AKT signal pathway.

## Conclusions

**Fig. 7:**
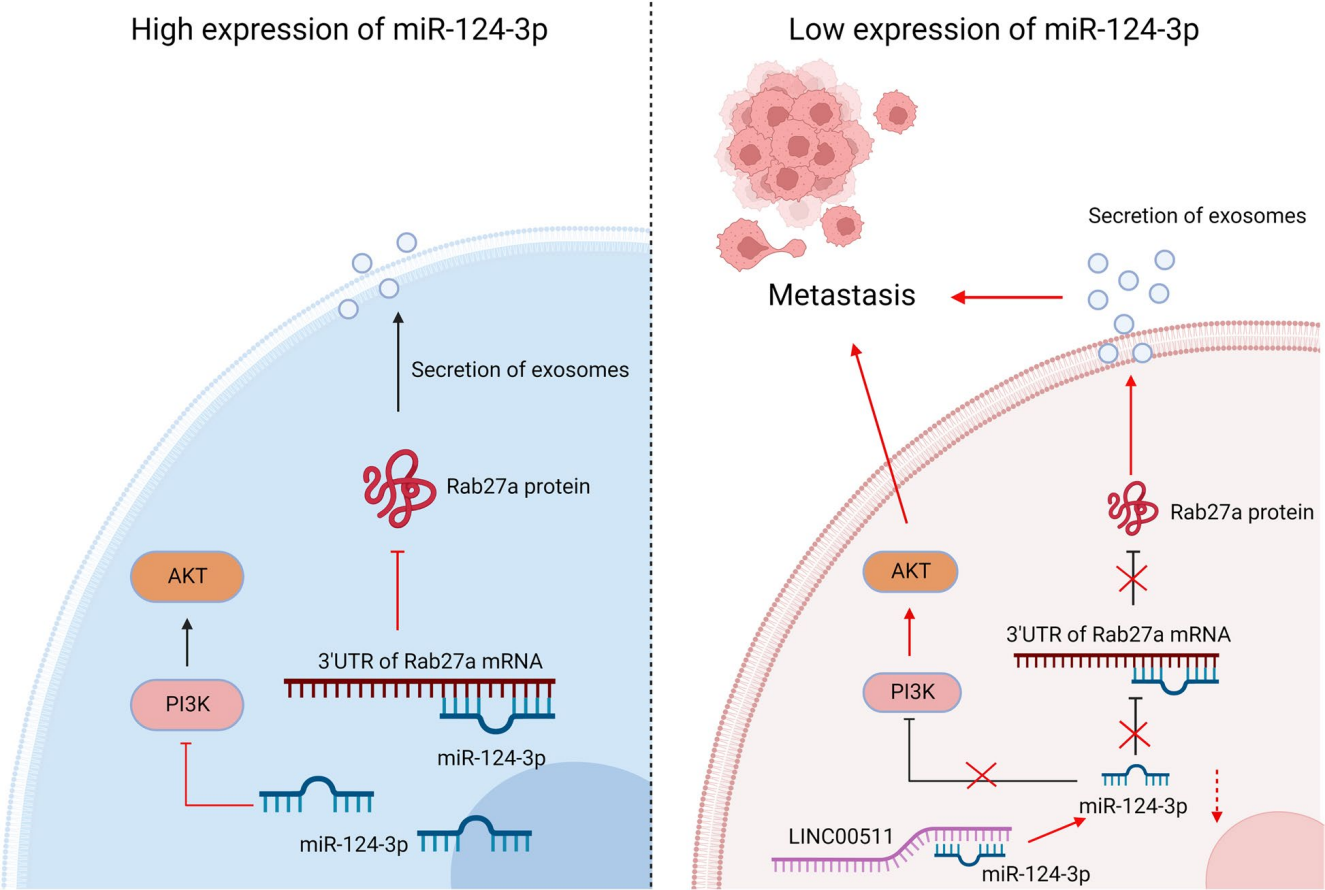
*miR-124-3p* prevents the metastasis of NSCLC via inhibiting the exosome secretion and PI3K/AKT signaling pathway

In this study, our results demonstrate that m*iR-124-3p* is an inhibitor of metastasis in NSCLC, and it can bind to the 3’UTR of Rab27a, thereby suppressing the translation process of Rab27a, leading to a reduction in exosome secretion It also inhibits the activation of PI3K/AKT signaling pathway, and either function of m*iR-124-3p* enables the repression of the metastasis in NSCLC. However, when the expression of *LINC00511* is elevated, it can function as a ceRNA of m*iR-124-3p*, thus the regulation of m*iR-124-3p* is suppressed, which promotes the metastasis of NSCLC (Fig. 7). These discoveries provide a better understanding of the metastasis in NSCLC and suggest a potential treatment for NSCLC.

## Supplementary Information

**Additional file 1: Fig. S1.** GW4869 inhibits NSCLC cells proliferation, invasion and migration through inhibition of exosome secretion. **Fig. S2.** *miR-124-3p* inhibits exosome secretion, uptake and migratory abilities of NCI-H1299 cells. **Fig. S3.** The correlation between *miR-124-3p* and *LINC00511*. **Fig. S4.** *miR-124-3p* inhibits tumor proliferation by targeting Rab27a.

**Additional file 2**: **Table S1.** General information of NSCLC patients. **Table S2.** The sequences of primers for qPCR. **Table S3.** The RNA-seq annotation between control and *miR-124* mimic group. **Table S4.** The statistical data of clone formation, wound healing, cell invasion and cell migration.

**Additional file 3.** Original images for Western Blots. Figure 1c Original images for Western Blots. Figure 4d Original images for Western Blots. Figure 5f Original images for Western Blots

## Data Availability

All data produced or analyzed in this study are contained in this published article and its supplementary information files. The datasets used and/or analyzed in this research are accessible by the corresponding authors upon reasonable request.

## Abbreviations

*3’UTR*: 3 ‘non-coding region
*BCA*: Bicinchoninic acid
*BM-MSCs*: Bone Marrow Mesenchymal Stromal Cells
*cAMP*: Cyclic adenosine monophosphate
*CCK-8*: Cell Counting Kit-8
*CDK4*: Cyclin dependent kinase 4
*CDK6*: Cyclin dependent kinase 6
*ceRNA*: Competing endogenous RNA
*FBS*: Fetal Bovine Serum
*IHC*: Immunohistochemistry
*LUAD*: Lung adenocarcinoma
*LUSC*: Lung squamous cell carcinoma
*NSCLC*: Non-small cell lung cancer
*NTA*: Nanoparticle Tracking Analysis
*PI3K/AKT*: Phosphatidylinositol-3-kinase/AKT
*PVDF*: Polyvinylidene fluoride
*qPCR*: Quantitative real-time PCR
*TEM*: Transmission electron microscope
*TSG101*: Tumor Susceptibility 101
*DNMT3B*: DNA methyltransferase 3B
*TME*: Tumor microenvironment
