# Supplementary material for: MiR-124-3p impedes the metastasis of non-small cell lung cancer via extracellular exosome transport and intracellular PI3K/AKT signaling
Source: Biomark Res. 2023 Jan 4;11:1. doi: 10.1186/s40364-022-00441-w (PMC9811783; doi:10.1186/s40364-022-00441-w)
Supplement: Supplementary file 1 — Additional file 1: Fig. S1. GW4869 inhibits NSCLC cells proliferation, invasion and migration through inhibition of exosome secretion. Fig. S2. miR-124-3p inhibits exosome secretion, uptake and migratory abilities of NCI-H1299 cells. Fig. S3. The correlation between miR-124-3p and LINC00511. Fig. S4. miR-124-3p inhibits tumor proliferation by targeting Rab27a. [file 40364_2022_441_MOESM1_ESM.pdf]

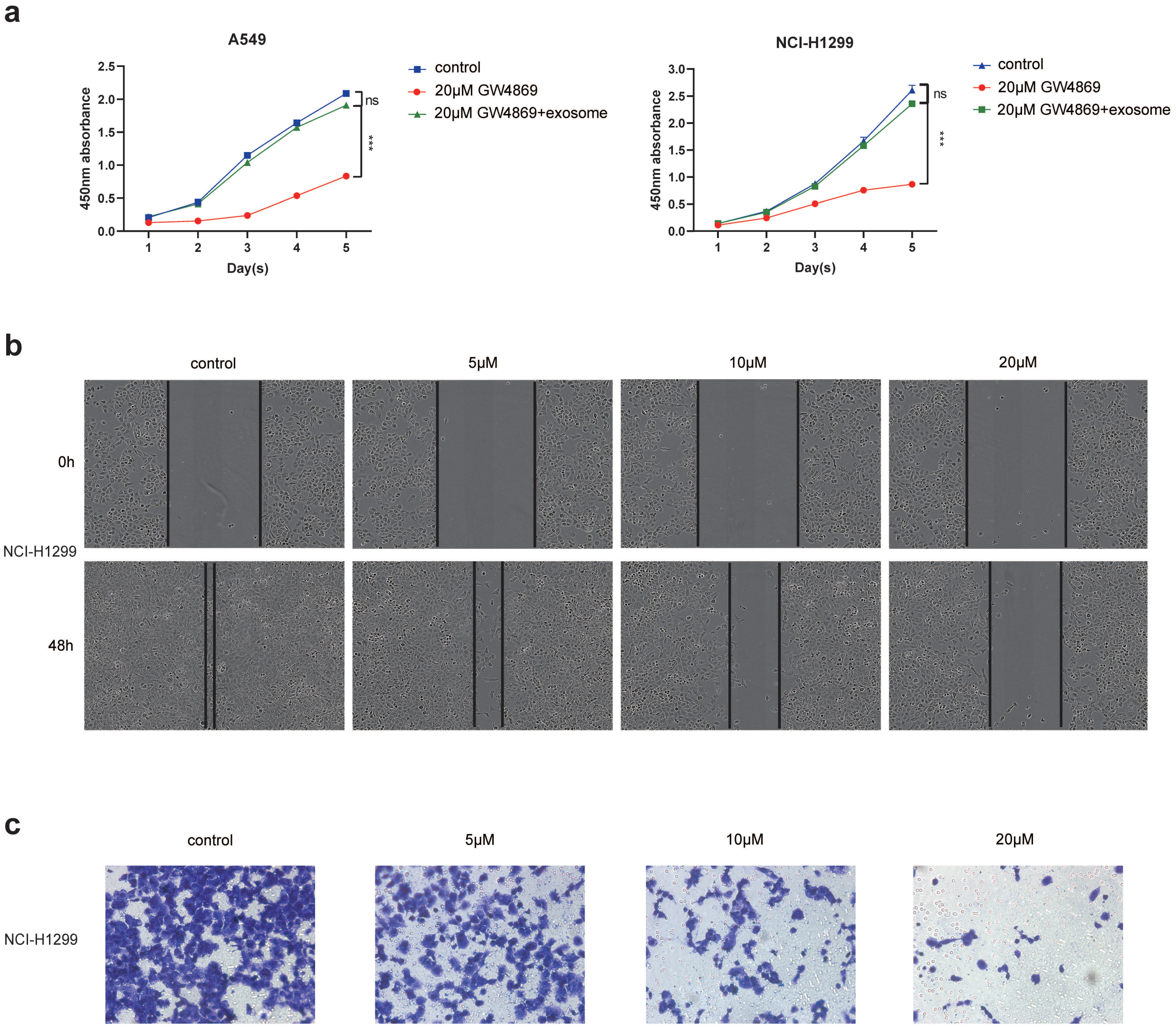

Figure S1. GW4869 inhibits NSCLC cells proliferation, invasion and migration through inhibition of exosome secretion.

(a) Growth curve of NSCLC cells treated with 20μM GW4869 and 20μM GW4869+exosome. **(b)**Images of wound healing assays of NCI-1299 cells transfected with different concentrations of GW4869. **(c)** Images of transwell assays performed in NCI-H1299 cells transfected with different concentrations of GW4869. Data are shown as the mean ± SD. *P < 0.01, **P < 0.001, ***P < 0.0001, and ****P < 0.00001.

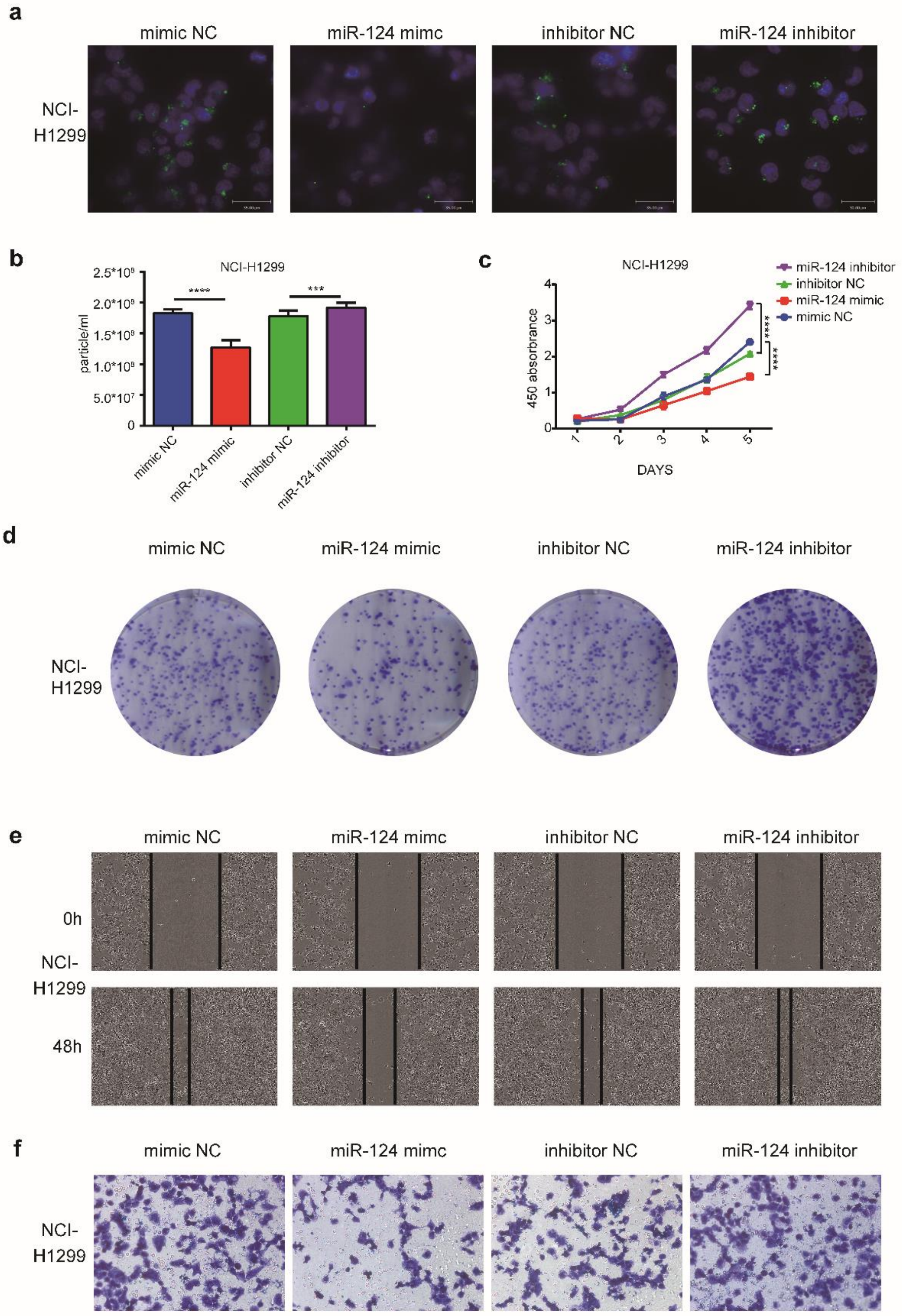
a
mimic NC
miR-124 mimc
inhibitor NC
miR-124 inhibitor
NCI-H1299
b
NCI-H1299
$2.5*10^{8}$
$2.0*10^{8}$
$1.5*10^{8}$
$1.0*10^{8}$
$5.0*10^{7}$
0
particle/ml
****
***
mimic NC
miR-124 mimic
inhibitor NC
miR-124 inhibitor
c
NCI-H1299
450 absorbrance
DAYS
miR-124 inhibitor
inhibitor NC
miR-124 mimic
mimic NC
****
****
d
mimic NC
miR-124 mimic
inhibitor NC
miR-124 inhibitor
NCI-H1299
e
mimic NC
miR-124 mimc
inhibitor NC
miR-124 inhibitor
0h
NCI-H1299
48h
f
mimic NC
miR-124 mimc
inhibitor NC
miR-124 inhibitor
NCI-H1299

Figure S2. miR-124-3p inhibits exosome secretion, uptake and migratory abilities of NCI-H1299 cells

(a) Concentration of exosomes in medium from A549 cells transfected with miR-124-3p mimic or inhibitor. **(b)**Images of the uptake of exosomes fluorescently labeled with PKH67 (green) by A549 after incubation for 24 hours. Images were acquired under confocal microscopy. **(c)** Growth curves generated with OD450 data with the CCK8 assay for A549 cells transfected with miR-124-3p mimic or inhibitor. **(d)** Images of colony forming assays for A549 cells transfected with miR-124-3p mimic or inhibitor. Cells were fixed and stained with crystal violet. **(e)** Images of wound healing assays of A549 cells transfected with miR-124-3p mimic or inhibitor. **(f)** Images of transwell assays performed with A549 cells transfected with miR-124-3p mimic or inhibitor. Cells were fixed and stained with crystal violet. Data are shown as the mean ± SD. *P < 0.01, **P < 0.001, ***P < 0.0001, and ****P < 0.00001.

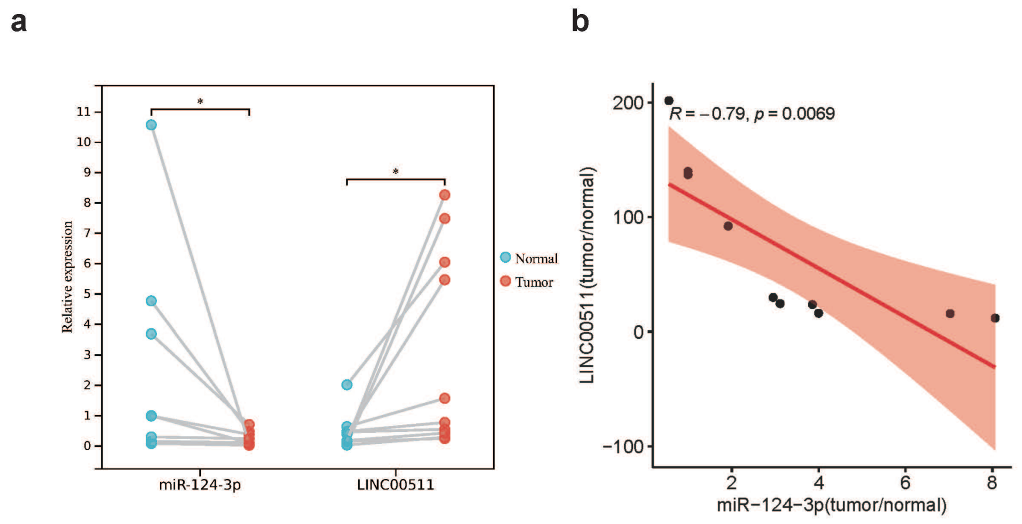

Figure S3. The correlation between miR-124-3p and LINC00511

(a) The relative expression of miR-124-3p and LINC00511 in normal or tumor tissues of NSCLC.**(b)** Images of the correlation analysis between miR-124-3p and LINC00511 in NSCLC. Data are shown as the mean ± SD. *P < 0.01, **P < 0.001, ***P < 0.0001, and ****P < 0.00001.

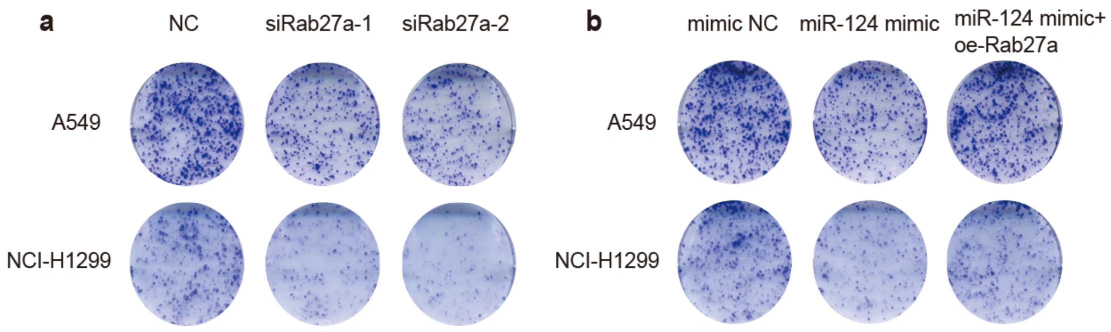

Figure S4. *miR-124-3p* inhibits tumor proliferation by targeting Rab27a

(a) Images of colony formation assays performed on A549 and NCI-H1299 cells transfected with siRNA against Rab27a, si-Rab27a. **(b)** Images of colony forming assays performed on A549 and NCI-H1299 cells transfected with miR-124-3p mimic or miR-124-3p mimic + oe-Rab27a.
