# Supplementary figures and images for: MiR-124-3p impedes the metastasis of non-small cell lung cancer via extracellular exosome transport and intracellular PI3K/AKT signaling
Source: Biomark Res. 2023 Jan 4;11:1. doi: 10.1186/s40364-022-00441-w (PMC9811783; doi:10.1186/s40364-022-00441-w)

## Original images for Western Blots

Figure 1c

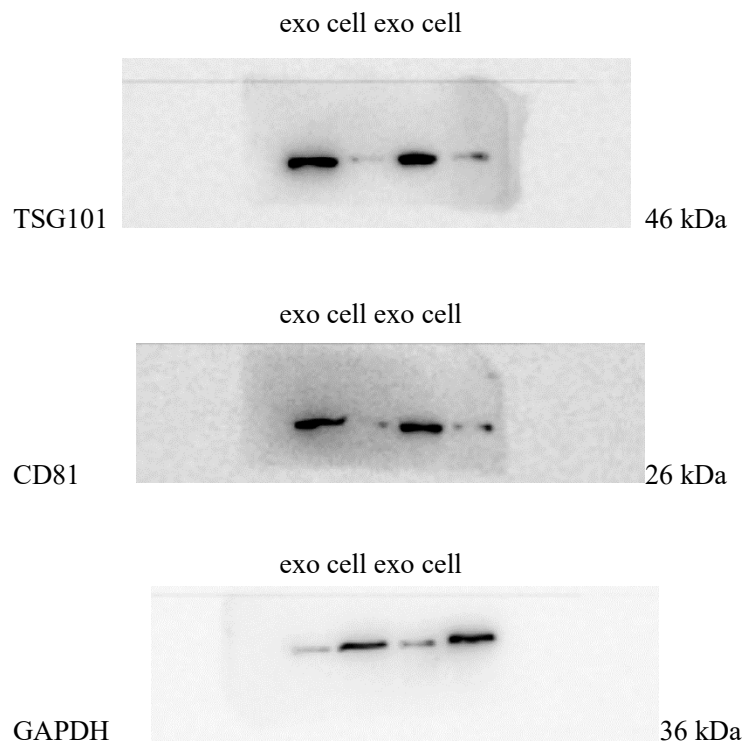

Figure 4d

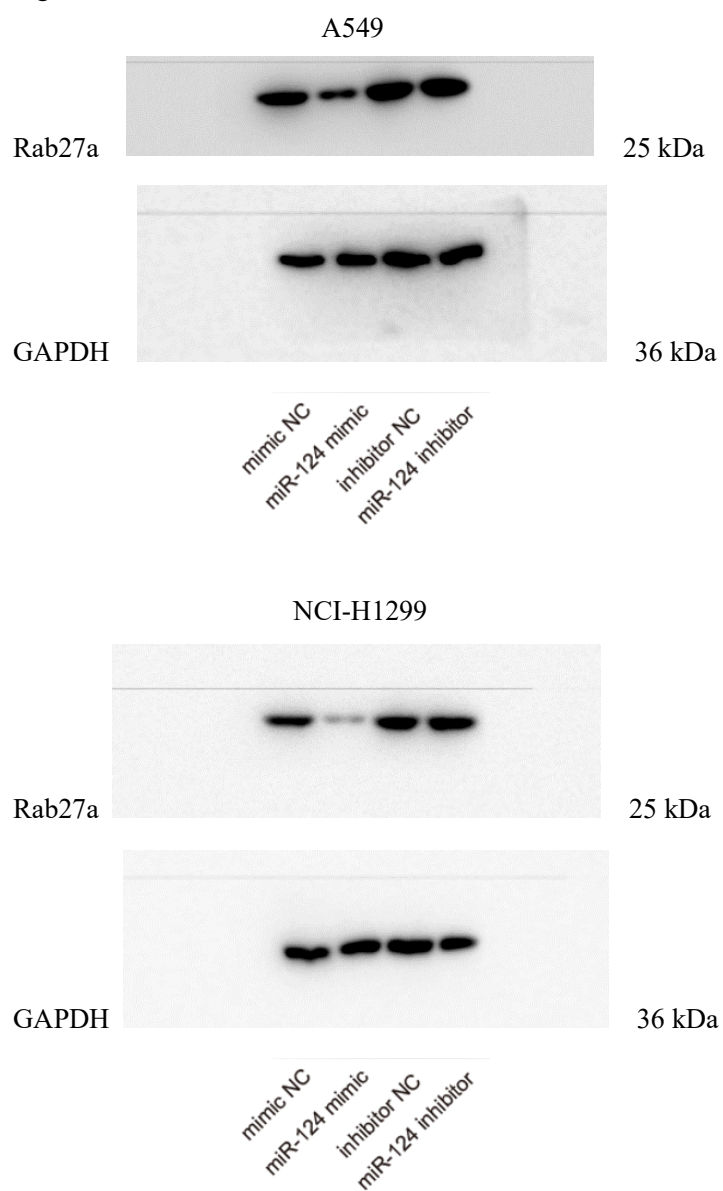

Figure 5f

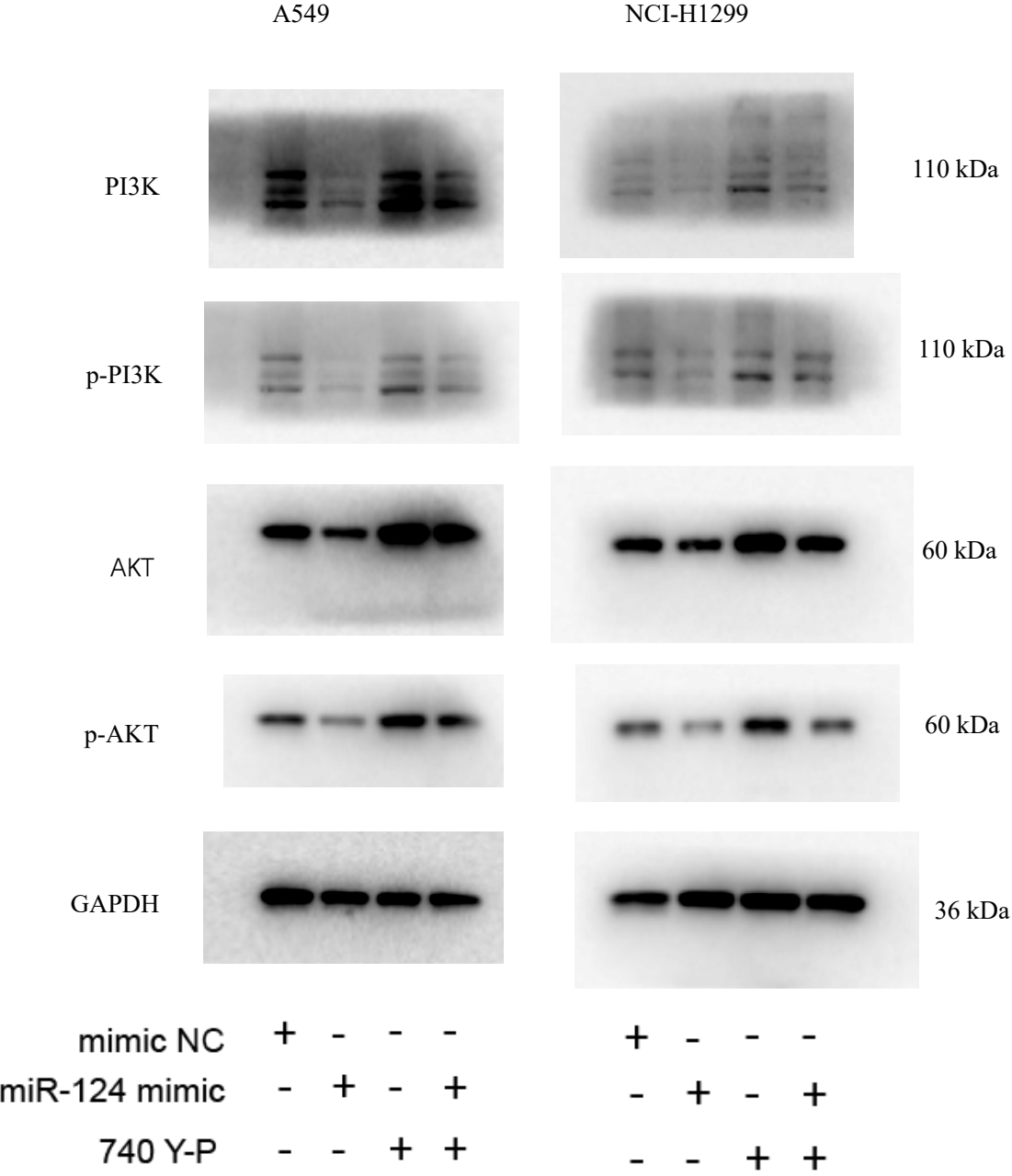

Supplement: Supplementary file 3 — Additional file 3. Original images for Western Blots. Figure 1c Original images for Western Blots. Figure 4d Original images for Western Blots. Figure 5f Original images for Western Blots [file 40364_2022_441_MOESM3_ESM.pdf]
